# Molecular Determinants of Agonist Selectivity in Glutamate-Gated Chloride Channels Which Likely Explain the Agonist Selectivity of the Vertebrate Glycine and GABA_A_-ρ Receptors

**DOI:** 10.1371/journal.pone.0108458

**Published:** 2014-09-26

**Authors:** Thomas Blarre, Hugues-Olivier Bertrand, Francine C. Acher, JacSue Kehoe

**Affiliations:** 1 Laboratoire de Chimie et Biochimie Pharmacologiques et Toxicologiques, UMR8601-Centre National de la Recherche Scientific, Université Paris Descartes, Sorbonne Paris Cité, Paris, France; 2 Accelrys, Orsay, France; 3 Laboratoire de Physiologie Cérébrale, UMR8118-Centre National de la Recherche Scientifique, Université Paris Descartes, Paris, France; Dalhousie University, Canada

## Abstract

Orthologous Cys-loop glutamate-gated chloride channels (GluClR’s) have been cloned and described electrophysiologically and pharmacologically in arthropods and nematodes (both members of the invertebrate ecdysozoan superphylum). Recently, GluClR’s from *Aplysia californica* (a mollusc from the lophotrochozoan superphylum) have been cloned and similarly studied. In spite of sharing a common function, the ecdysozoan and lophotrochozoan receptors have been shown by phylogenetic analyses to have evolved independently. The recent crystallization of the GluClR from *C. elegans* revealed the binding pocket of the nematode receptor. An alignment of the protein sequences of the nematode and molluscan GluClRs showed that the *Aplysia* receptor does not contain all of the residues defining the binding mode of the ecdysozoan receptor. That the two receptors have slightly different binding modes is not surprising since earlier electrophysiological and pharmacological experiments had suggested that they were differentially responsive to certain agonists. Knowledge of the structure of the *C. elegans* GluClR has permitted us to generate a homology model of the binding pocket of the *Aplysia* receptor. We have analyzed the differences between the two binding modes and evaluated the relative significance of their non-common residues. We have compared the GluClRs electrophysiologically and pharmacologically and we have used site-directed mutagenesis on both receptor types to test predictions made from the model. Finally, we propose an explanation derived from the model for why the nematode receptors are gated only by glutamate, whereas the molluscan receptors can also be activated by β-alanine, GABA and taurine. Like the *Aplysia* receptor, the vertebrate glycine and GABA_A_-ρ receptors also respond to these other agonists. An alignment of the sequences of the molluscan and vertebrate receptors shows that the reasons we have given for the ability of the other agonists to activate the *Aplysia* receptor also explain the agonist profile seen in the glycine and GABA_A_-ρ receptors.

## Introduction

Rapid synaptic transmission in the nervous system is mediated by a very large and diverse family of ligand-gated ion channels (LGICs). There are three major divisions in the family based on channel structure: trimeric, tetrameric and pentameric. Two types of trimeric LGIC have been described, both of which are cationic channels: the P2X ATP-activated channel [1] and the proton-activated acid-sensing ion channels (ASIC) [2].

The major excitatory LGICs in the central nervous system are glutamate-gated cationic channels [3] that account for all tetrameric LGICs. One subtype of glutamate receptors - the NMDA receptors - can only be activated in the presence of a co-agonist (glycine, serine or alanine [3]) which binds to an independent site.

The other major group of LGICs are of pentameric structure, and, in eukaryotes, are characterized by a highly conserved cysteine loop that is found in the extracellular N-terminal domain^.^ [4]–[7]. This characteristic has led to their being labeled “cys-loop receptors”. The pentameric receptors, unlike those of the trimeric and tetrameric groups, include receptors for many different neurotransmitters: ACh [8], GABA [9], glycine [10], serotonin [11], glutamate [12]–[15], histamine [16]–[17] dopamine and tyramine [18], and other LGICs that are inhibited [19] or gated by protons [20]–[21], or gated by Zn^2+^ [22]. Furthermore, a given transmitter type can be associated with both a cationic and an anionic channel (e.g., ACh [23], GABA [9], [24], 5-HT [11], [25]). Most of this diversity is found in invertebrates [12]–[27].

The pentameric receptors can, themselves, be further divided into two distinct groups: those containing only the cys loop which is associated with all eukaryote pentameric receptors and those which have been labeled 2-cys-loop receptors [26] since they contain an additional cys loop in the N-terminal domain.

The 1-cys loop receptors include both excitatory (cationic) and inhibitory (anionic) receptors whereas all 2-cys loop LGICs discovered to date are anionic. The glycine receptor [10] is the best known example of the 2-cys loop receptors, and the only vertebrate one. In invertebrates, 2-cys-loop receptors have been found that are activated by either glutamate [12]–[15] or histamine [16]–[17] and others which are inhibited [19] or gated [20] by protons.

Until recently the only invertebrate 2-cys loop receptors that had been cloned, expressed, and for which a function had been determined were from nematodes and arthropods. Those two phyla represent only one of the two major superphyla of invertebrates - the ecdysozoa. In 2009 the cloning of glutamate-gated chloride channels from the mollusc *Aplysia californica* [14] provided sequences of 2-cys loop receptors from the other major group of invertebrates - the lophotrochozoa, consisting principally of molluscs and annelids.

A phylogenetic analysis was performed [14] on the ecdysozoan 2-cys-loop receptors described above, the mammalian glycine receptor, the *Aplysia* glutamate receptors and homologs of the *Aplysia* receptors taken from genomes recently sequenced by the Joint Genome Institute from two other lophotrochozoa: the mollusc *Lottia gigantea* and the annelid, *Capitella teleta*. The analysis revealed that the glutamate receptors from nematodes and arthropods (ecdysozoa) are, phylogenetically speaking, independent of the glutamate receptors from the molluscs and annelids (lophotrochozoa). Either the receptors from these two groups share a common ancestral glutamate-sensitive protein from the deep roots of the metazoan lineage, or the binding of glutamate to these two receptor types results from convergent evolution. A similar phylogenetic independence of nematode and molluscan 1-cys-loop ACh-gated chloride channels has been noted [26]–[28].

The conclusion that the *Aplysia* and nematode glutamate-gated chloride channels were not orthologs was not a complete surprise. Electrophysiological studies of glutamate-gated chloride channels in *Aplysia* neurons and of heterologously-expressed glutamate receptor subunits from *C. elegans* had already suggested the existence of pharmacological differences in the receptors from the different phyla. Whereas GABA and β-alanine had been shown to activate the *Aplysia* neurons bearing the glutamate receptor [29]–[30], no sign of a response to GABA was seen from the heterologously expressed nematode receptor [31]–[32], although the GABA concentrations used were relatively low given the high EC_50_’s for glutamate that were recorded for those receptors.

In 2011 the crystalization by Hibbs and Gouaux [33] of one of the alpha subunits of the 2-cys-loop glutamate receptor from *C. elegans* defined the binding mode of that receptor. An alignment of the protein sequence of the nematode glutamate receptor with that from the *Aplysia* reveals that the *Aplysia* receptor does not contain, in homologous positions, all of the residues belonging to the binding pocket of the nematode receptor.

Knowing the structure of the binding pocket of the ecdysozoan receptor has made it possible for us to predict, by homology modeling, the binding mode of one of the two glutamate-gated chloride channels in *Aplysia* and to confirm, by site-directed mutagenesis, predictions drawn from the model. Heterologous expression of both the *C. elegans* and *Aplysia* receptors in the same expression system has made it possible to establish certain distinctive pharmacological characteristics of the two receptor types and to suggest, in light of the model, a possible reason for these differences.

## Methods

### Cell culture, mutagenesis and electrophysiology

#### Cell culture and transfection

Chinese Hamster Ovary (CHO-K1) cells were obtained from the American Type Tissue Culture Collection (ATCC, Molsheim, France) and maintained in HAM-F12 nutrient mix+GluaMAX supplement (Life Technologies) after adding 10% fetal bovine serum (Gibco 10500) and 1% PenStrep (Gibco 15140). Cells were plated in 35 mm Falcon easy grip dishes and were split one to three days prior to transfection directly into new 35 mm dishes with 2 ml of medium. On the day of, but prior to, transfection the old medium was replaced by 1 ml of fresh medium. cDNAs were introduced into the CHO cells using the transfection agent Exgen 500. The protocol prescribed by Thermo Scientific was adapted for use with 35 mm dishes and for the amount of cDNA used in each transfection. 1 µg of a WT or mutated glutamate receptor cDNA (see above) was co-transfected with 1 µg of Green Fluorescent Protein (GFP). Our procedures, however, included one exception to the Exgen protocol: the transfection dish contained only 1 ml of medium for the first 80 min of exposure to the DNA-Exgen mix. At the end of that time period, one more ml of medium was added and the Exgen-cDNA containing milieu was left in the dish, as suggested by the Exgen protocol. The fluorescent cells were studied, electrophysiologically, 24–30 hours after the initiation of transfection.

#### Fast perfusion system and electrophysiological recording

A modified perfusion system from ALA Scientific Instruments, with an 8-channel solenoid valve manifold (VC-3-8), was used for applying a constant rapid flow of solution directly onto the cell under study. At most six syringe and tube assemblies (PE-20) containing various agonists and/or various agonist concentrations fed into a Warner Instruments ML-6 miniature manifold from which the control external solution or agonist-containing solution exited through a BD Microlance hypodermic needle (25 g, 25 mm, 1″ regular wall) approximately 30–50 µM from the cell. All solutions were gravity fed from these syringes which were approximately ≈50 cm above the preparation. An agonist was typically applied for 1 sec followed by a 3-min flow of control solution through the same tube (see above). In addition, the dish of transfected cells was continuously superfused with external control solution through an independent tube.

Whole-cell recordings were conducted at room temperature (20–25°C) using an EPC-9 amplifier (HEKA Elektronik, Germany). The external solution contained (in mM): NaCl 140, KCl 5, CaCl_2_ 2, MgCl_2_ 1, HEPES 20, and glucose 25 brought to pH 7.4 with NaOH; ≈310–320 mOsm. The patch pipette solution contained (in mM): KCl 140, MgCl_2_ 2, MgATP 2, NaGTP 0.4, HEPES/KOH 10, BAPTA/KOH 20; pH 7.3; ≈290 mOsm. Pipettes were pulled from borosilicate glass capillaries (with filament) on a vertical pipette puller (L/M-3P-A, List-Medical, Darmstadt, Germany) and had resistances of 3–6 MOhms. Only cells with an input resistance over 300 MOhms were used in the experiments reported here.

#### The choice of glutamate-gated chloride channels from *C. elegans* and *Aplysia californica*

For the electrophysiological and pharmacological evaluation of the glutamate-gated chloride channel from *C. elegans* we chose not to use the GluClα1 subunit [12] – a slight modification of which was crystalized by Hibbs and Gouaux [33]. Neither GluClα1 nor its modified version, GluCl_Cryst_, respond to glutamate without a preactivation by ivermectin [12], [33] due to a failure of that subunit to couple agonist binding to channel gating [31]. Consequently, we have chosen another alpha subunit from *C. elegans* - GluClα2b [32] (accession number CAA04170) - which does respond directly to glutamate. GluClα2b, like the other alpha subunits of the glutamate-gated chloride channels of nematodes and arthropods, has the same binding residues as does the crystalized receptor. The glutamate-gated chloride channel from *Aplysia* that was used for both the homology model and the electrophysiological and pharmacological comparison was GluCl*Ac*2 [14] (accession number NP_001191520).

#### Site-directed mutagenesis

The QuickChange site-directed mutagenesis kit by Stratagene (Agilent Technologies) was used for mutating specific residues in GluCl*Ac*2 and in GluClα2b. In the event that two or three residues were to be mutated, the mutations were performed successively: i.e., an additional mutation was made on an already mutated receptor. All mutations were confirmed by sequencing.

### Homology modeling, molecular dynamics and docking

#### Homology modeling

All calculations were performed in Discovery Studio 3.5 (Accelrys Software Inc., San Diego, CA).

#### Homology model for glutamate-binding domain of GluCl*Ac*1 and GluCl*Ac*2

The sequence and crystallographic structure of the glutamate-binding domain of the *C. elegans* GluCl_Cryst_ receptor were retrieved from the PDB (PDB code: 3RIF). The GluCl receptors, like the glycine receptor, are pentamers. Glutamate binds between two subunits of this pentamer. Hence, to accurately analyze glutamate binding in *C. elegans* and *Aplysia*, the sequence, structure and models of two subunits were always considered.

The sequence of the glutamate-binding domain of *Aplysia* GluCl*Ac*1 and GluCl*Ac*2 receptors were retrieved from the NCBI database (NCBI codes: NP_001191519 and NP_001191520 respectively). The sequences of GluClα1, GluCl*Ac*2 and GluCl_Cryst_ were aligned using the Align123 algorithm [34], and the resulting sequence alignment was then further used for comparative modeling.

Models of GluCl*Ac*1 and GluCl*Ac*2 (two adjacent subunits of each of the two receptors) were generated using MODELER [35]. For each receptor, 100 models were generated but only the ten best with respect to lowest PDF Energy (as provided by MODELER) were considered for further analysis.

Subsequently we calculated their Profiles_3D scores [36]–[37] and the percentage of amino-acids in disallowed regions of the Ramachandran plot. For each receptor, the model exhibiting the best consensus between the three properties cited above was chosen for molecular dynamics.

During all the model building process, the glutamate present in the template was kept and transferred into the models. As a result, glutamate was already positioned for subsequent calculations.

#### Molecular dynamics

Protein-ligand interactions (GluCl*Ac*1-glutamate and GluCl*Ac*2-glutamate) were further optimized by 2 ns molecular dynamics using CHARMm [38]. Once the trajectory was equilibrated, snapshots of the trajectory were analyzed in terms of protein-ligand contacts and the selected ones were submitted to a final CHARMm-based energy minimization.

#### Mutated GluCl*Ac*1 receptor

The protein sequence of GluCl*Ac*1 was modified (S95R and K158Y mutations were introduced) and the above mentioned protocol was performed on this sequence.

#### Docking of additional ligands

For each docking experiment, the ligand was initially positioned in the binding site using CDOCKER. CDOCKER uses a CHARMm-based molecular dynamics (MD) protocol to dock ligands into a receptor binding site [39]. Random ligand conformations were generated using high-temperature MD. The conformations were then translated into the binding site and candidate poses were then created using random rigid-body rotations followed by simulated annealing. A final CHARMm-based energy minimization was then used to refine the ligand poses.

## Results and Discussion

### Electrophysiological and pharmacological evaluation of GluClα2b (*C. elegans*) and GluClAc2 (*Aplysia californica*)

#### Differential desensitization properties of the two receptors

Under the conditions used here to study the responses of the two receptors to glutamate, the GluCl*Ac*2 receptor was found to desensitize much more rapidly than does the GluClα2b receptor. The response of the GluCl*Ac*2 receptor to a 1-sec application of 1 mM glutamate reaches a maximum rapidly and, by the end of the 1-sec application, is already reduced to 27% of its maximum amplitude, whereas the response of GluClα2b is only reduced to 63% of its maximum at that time (One-way ANOVA, p<.0001, n = 10 and SE = 0.04 for both evaluations). This differential desensitization is also seen when evaluating the constancy of the response amplitude over repeated applications: a 1-min interval between 1-sec applications of 1 mM glutamate is insufficient for recovering a constant response amplitude from the GluCl*Ac*2 receptor, whereas an essentially identical response can be obtained from GluClα2b receptor with that protocol. Because of this difference a minimum of a 3-min interstimulus interval was always used when analyzing the properties of the GluCl*Ac*2 receptor (see Methods).

#### Sensitivity to glutamate of the two receptor types

The EC_50_ of the glutamate response of GluCl*Ac*2 expressed in CHO cells has already been estimated to be 196 µM [14]. In contrast, the only published EC_50_ for GluClα2b was obtained by expression in *Xenopus* oocytes [32] and was found to be 2 mM. To examine the sensitivity of the two receptor types in the same expression and perfusion systems we have not attempted to do full concentration-response curves, but have compared their responses only to 100 mM, 1 mM and 10 mM glutamate. In GluClα2b, the response to 1 mM was 94% of that to 10 mM (n = 14, SE = 2.1); in GluCl*Ac*2, 95.5% (n = 17, SE = 1.8) (one way ANOVA, p = 0.6). These results suggest that both receptors have, in our system, reached saturation at 1 mM.

On the other hand, a difference in the sensitivity of the two receptors to 100 µM glutamate was observed: in GluClα2b, the response to 100 µM was only 12.2% of that to 1 mM glutamate (n = 14, SE = 5); in GluCl*Ac*2, 57% (n = 14, S = 5) (one-way ANOVA, P<0.0001). This difference at lower concentrations reinforces the estimates of relative sensitivity of the two receptors drawn from published data obtained from different expression systems (see above).

#### Agonist specificity of the two receptor types

**Figure 1:**
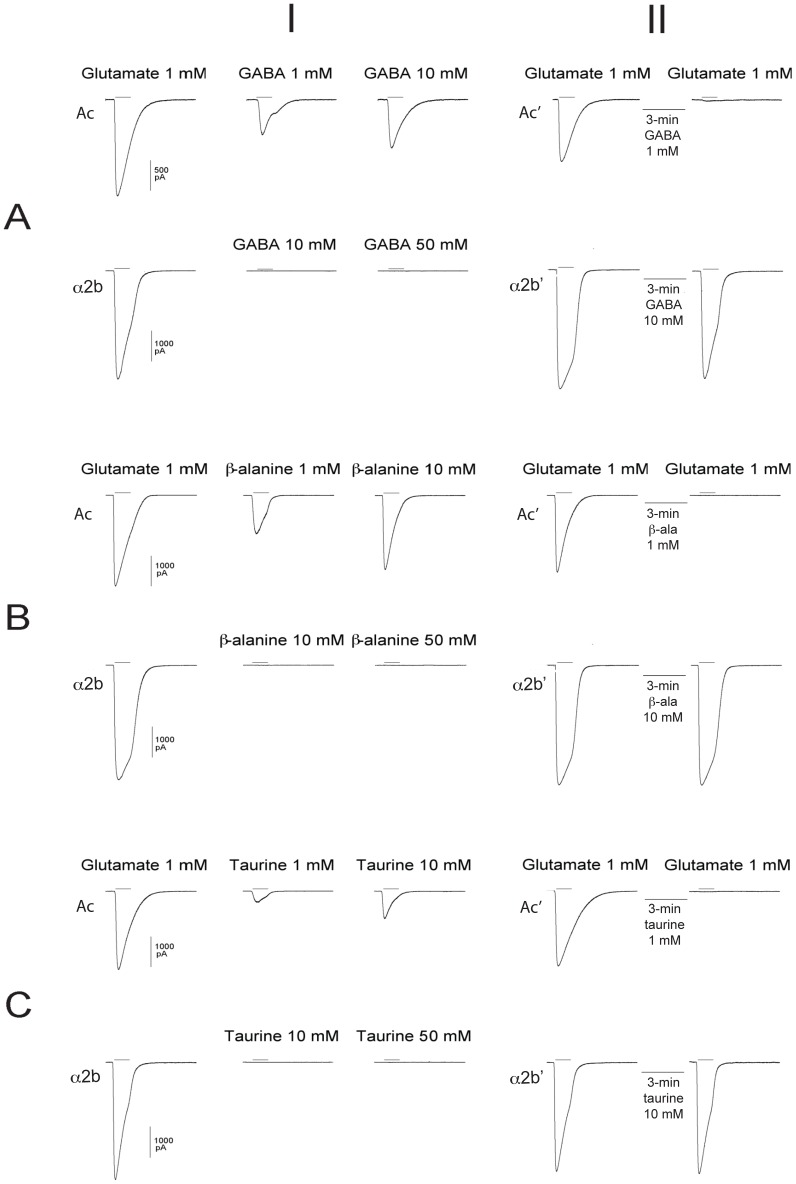
Differential sensitivity of GluCl*Ac*2 and GluClα2b receptors to glutamate, GABA, β-alanine and taurine. **Column labeled I:** Traces preceded by Ac: Responses of GluCl*Ac*2 to a 1-sec application of (A) 1 mM glutamate, 1 mM GABA and 10 mM GABA; (B) 1 mM glutamate, 1 mM β-alanine and 10 mM β-alanine; and (C) to 1 mM glutamate, 1 mM taurine and 10 mM taurine. Traces preceded by α2b: Responses of GluClα2b to a 1-sec application of (A) 1 mM glutamate, 10 mM GABA and 50 mM GABA; (B) 1 mM glutamate, 10 mM β-alanine and 50 mM β-alanine; and (C) 1 mM glutamate, 10 mM taurine and 50 mM taurine. **Column labeled II:** Traces preceded by Ac′: Responses of GluCl*Ac*2 to a 1-sec application of glutamate before and after a 3-min application of (A) 1 mM GABA; (B) 1 mM β-alanine, and (C) 1 mM taurine. Traces preceded by α2b: Responses of GluClα2b to a 1-sec application of glutamate before and after a 3-min application of (A) 10 mM GABA; (B) 10 mM β-alanine, and (C) 10 mM taurine. All applications of glutamate or other agonists were separated by a 3-min interval (during which the control solution bathed the cell), except in column II where a second agonist (GABA, β-alanine, or taurine) was applied during the 3-min interval separating glutamate applications.

The glutamate-gated chloride channels of *Aplysia* have been shown, both in situ [29]–[30] and in homomeric expression in CHO cells [14], to be activated by both GABA and β-alanine. Given the high EC_50_’s for the response to glutamate of the GluClα2b receptor expressed in *Xenopus oocytes* [32], the concentrations used to evaluate the sensitivity of that receptor subunit to GABA (the only other agonist tested) could have missed a low sensitivity to that amino acid. Consequently, we have tested here, under identical transfection, recording and fast perfusion conditions, the differential sensitivity of the two receptor types to GABA, β-alanine and taurine, all of which are known to be present in the mammalian nervous system and all of which activate the glycine and GABA_A_-ρ receptors [40]–[44]. A comparison of the responses of GluCl*Ac*2 and GluClα2b to glutamate and the other three agonists can be seen in Fig. 1.

The records labeled “Ac” in column I are typical recordings of the responses of GluCl*Ac*2 to 1 mM glutamate and to 1 and 10 mM of either GABA (A, Ac), β-alanine (B, Ac), or taurine (C, Ac). All of the latter three agonists elicited responses to both the 1 and 10 mM concentrations.

In contrast, the records labeled “α2b” in column I, which show the responses obtained from GluClα2b, reveal that, in spite of a robust response to 1 mM glutamate, no currents could be elicited by 10 or 50 mM of either GABA (A, α2b), β-alanine (B, α2b), or taurine (C, α2b).

A similar difference in the two receptor types was seen when we evaluated the ability of the three other agonists to desensitize or block the responses of the two receptors to glutamate. In the records in the right hand column of Fig. 1 (II), during the 3-min interval separating 1-sec applications of glutamate, one of the three other agonists (A, B, C) was applied. The records preceded by Ac′ are from the GluCl*Ac*2 receptor, and the 3-min application of 1 mM of the non-glutamate agonist (A, Ac′ GABA; B, Ac′ β-alanine; C, Ac′ taurine) can be seen to have completely eliminated the response to the second application of glutamate.

In contrast, in cells expressing the GluClα2b receptor (records preceded by α2b′), the glutamate response that immediately follows a 3-min application of a 10 times higher concentration (10 mM) of either GABA, β-alanine or taurine is essentially identical to that preceding the “desensitizing” agonist (see A, α2b′, B, α2b′ and C, α2b′, respectively).

These findings confirm the conclusion drawn from the experiments done in Xenopus oocytes [32], i.e. that, GABA does not gate the GluClα2b receptor. They also show that the same conclusion can be drawn concerning β-alanine and taurine.

### A comparison of the crystallographic structure of the *C. elegans* glutamate-gated chloride channel and the homology model of GluClAc2 derived therefrom

#### Analysis of the sequence alignment of GluCl_Cryst_ and GluCl*Ac*2

**Figure 2:**
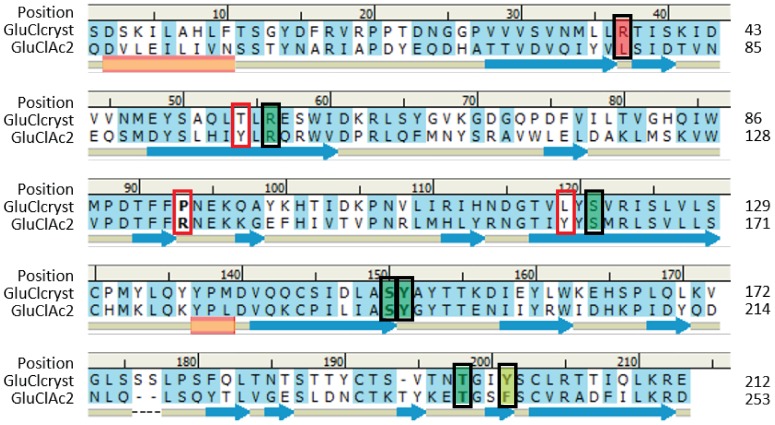
Alignment used for homology model building. Sequence alignment of residues of the two GluCl receptors: the first, a receptor from *C. elegans*, GluCl_Cryst_, which has been crystalized and which will serve as the template for the homology model of the second, GluCl*Ac*2, from the *Aplysia californica*. The numbers above the sequence represent the positions in the alignment based on the truncated receptor from GluCl_Cryst_. The last line describes the secondary structure of GluCl_Cryst_ with the blue arrows representing β-sheets and the orange tubes, the α-helices. Throughout the length of the sequences, a light blue highlighting indicates identical or similar residues. Residues binding glutamate in GluCl_Cryst_ are surrounded by a black rectangle. These positions are highlighted in green, yellow and red when the aligned residue in GluCl*Ac*2 is respectively identical, similar or different. Three additional positions are surrounded by a red rectangle and correspond to positions of residues binding glutamate only in the homology model of GluCl*Ac*2.

The similarities and differences between the nematode and *Aplysia* receptors are clearly represented in the sequence alignment shown in Fig. 2 which reveals that out of the seven binding residues identified in the crystallographic structure of the nematode receptor (each surrounded by a black rectangle), five are aligned with identical residues in GluCl*Ac*2 (colored in green in Fig. 2). One of the other binding residues of GluCl_Cryst_ (see residue position 201, colored in yellow) is aligned with a residue in GluCl*Ac*2 which could have a similar function (a tyrosine in GluCl_Cryst_ can be seen to be aligned with a phenylalanine in GluCl*Ac*2). The remaining binding residue of GluCl_Cryst_, in contrast, aligns with a residue in GluCl*Ac*2 that cannot assume a similar function: the arginine in GluCl_Cryst_ at position 37 is aligned with a leucine in the Glucl*Ac*2 receptor. These two residues are clearly functionally different, and they thereby permit us to envision some differences in the way the receptors are binding glutamate. The residues at position 37 are colored in red.

**Figure 3:**
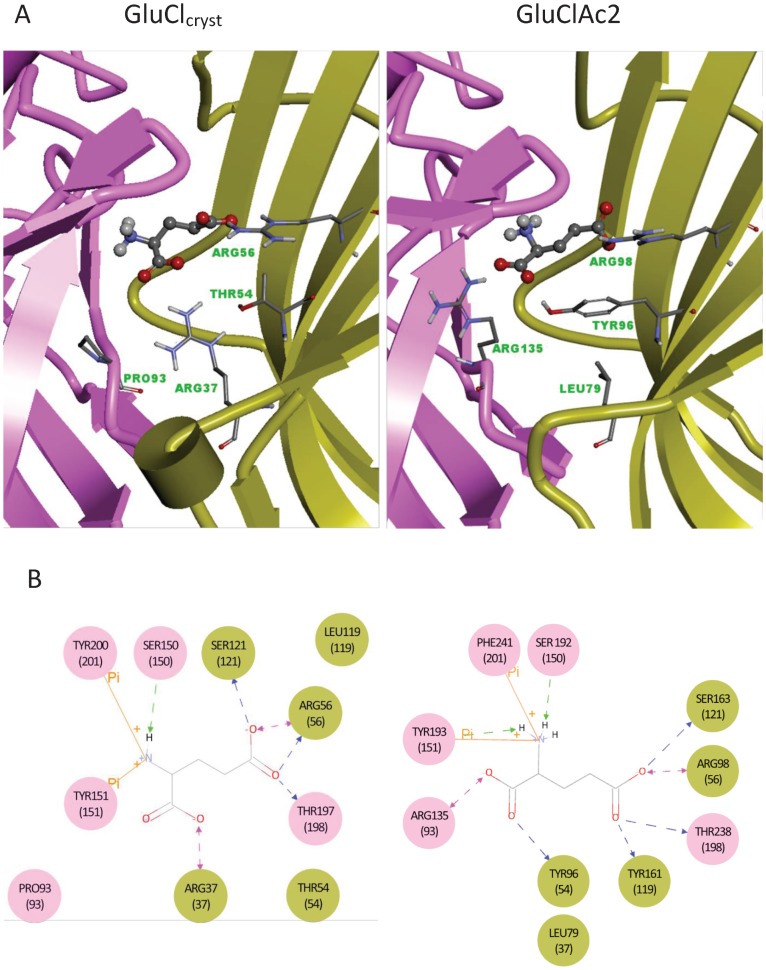
Glutamate bound to GluCl_Cryst_ and to the homology model of GluCl*Ac*2. A. 3D representation of the binding pocket of glutamate at the interface between the two subunits of the crystallographic structure of GluCl_Cryst_ (Left) and the model of GluCl*Ac*2 (Right). The Principal and Complementary subunits are displayed in violet and green/yellow, respectively. The bound glutamate is represented by a ball and stick display. The residues for four important positions in the alignment are represented as sticks, and their names and residue numbers are written in green. B. 2D diagram representing the interactions between glutamate and each of the two receptors: GluCl_Cryst_ (Left) and GluCl*Ac*2 (Right) as displayed in 3A. Glutamate is represented in lines, and adopts two different conformations reflecting the respective bindings at the two receptors. Only the polar hydrogens that are involved in interactions with the receptor are explicitly represented. Residues are depicted as circles in which the residue type, number and position (the latter in parentheses) are written on a colored background which indicates the subunit to which the residue belongs (see Fig. 3A). Backbone and side chain hydrogen bonds are represented by green and blue arrows, respectively. Salt bridges are represented by violet arrows and π interactions are represented by orange lines. Atom color code: carbon gray, oxygen red, nitrogen blue, hydogens white in A, black in B.

Three additional residues surrounded by red rectangles in Fig. 2 will be discussed only after the description of Fig. 3 which presents the proposed binding pocket of GluCl*Ac*2 predicted by the homology model. Note that none of the aligned residues surrounded by a red rectangle are identical, none are colored, and one of each of the three pairs of aligned residues belongs to the binding pocket of GluCl*Ac*2.

#### Crystallographic structure of GluCl_Cryst_ and the homology model of GluCl*Ac*2

The homology model of GluCl*Ac*2 (see Materials and Methods) along with the crystallographic structure of GluCl_Cryst_ has made it possible to compare the different amino acids interacting with glutamate in the two receptors.

#### Comparison of the binding modes of the two receptors

The GluCl_Cryst_ receptor is a pentamer, and Hibbs and Gouaux [33] have confirmed that glutamate binds between two adjacent subunits: the Principal (P) and Complementary (C) subunits as defined by Corringer et al. [45]. The relevant subunits with which the residues in Fig. 2 are associated have been indicated in the figure legend, and these subunits are represented schematically in Fig. 3.

The binding pockets of the GluCl_Cryst_ and GluCl*Ac*2 receptors, respectively, are represented in Fig. 3A with four of the important residues cited above shown as sticks associated with the Principal or Complementary subunit (colored in violet or green/yellow, respectively) to which each belongs.

**Figure 4:**
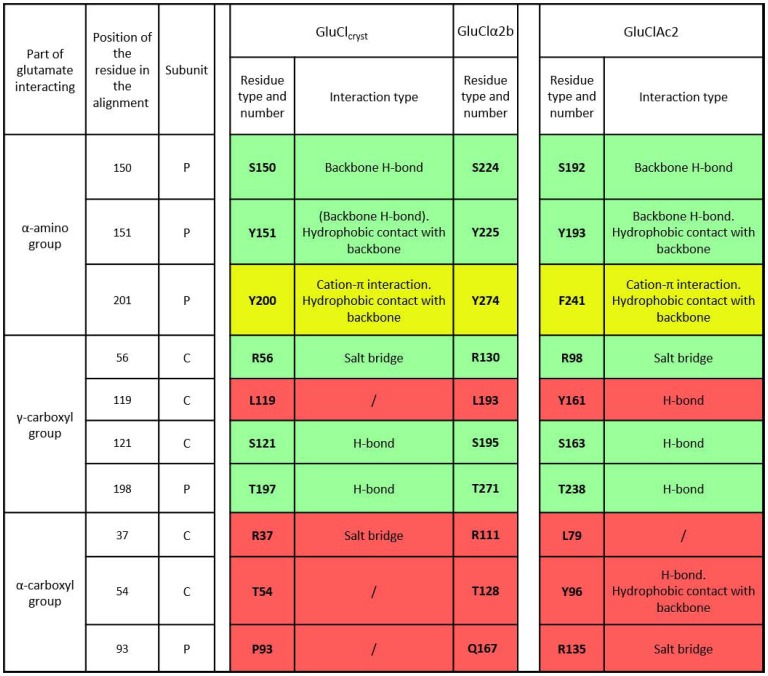
Summary of the interactions of glutamate in GluCl_Cryst_ and GluCl*Ac*2. List of residues (as identified from the crystallographic structure of GluCl_Cryst_ and predicted by the GluCl*Ac*2 homology model) accompanied by their position number in the alignment of Fig. 2 and a description of the type of interactions in which they are involved. Residue numbers are given for the crystalized receptor (GluCl_Cryst_), the receptor used in the electrophysiological experiments (GluClα2b), and the receptor that figures in the homology model (GluCl*Ac*2) from left to right, respectively. Rows in green, yellow and red indicate identical, similar and different aligned residues, respectively.

As can be seen in Figures 3 and 4, the serine and the tyrosine that are seen in both receptors at positions 150 and 151, respectively, bind the α-amino group directly, as do the tyrosine and phenylalanine residues seen at position 201 in the GluCl_Cryst_ and GluCl*Ac*2 receptors, respectively. The two aromatic rings of residues at positions 151 and 201 each make a cation-π interaction with the α-amino group of the ligand (Figures 3B and 4). Likewise, the three residues binding the γ-carboxyl group of GluCl_Cryst_ (at positions 56, 121 and 198) are the same in GluCl*A*c2 (see Figures 3B and 4).

The homology model of GluCl*Ac*2 (Fig. 3B) proposes an additional H-bond between the γ-carboxyl group of glutamate and residue Y161 (position 119, surrounded in red in Fig. 2) that is not present in the crystallographic structure of GluCl_Cryst_. However, it will be shown below that Y161 is not an essential residue in GluCl*Ac*2.

The major differences in the two binding pockets reside in the binding of the two receptors to the α-carboxyl group of the ligand. As shown in Fig. 3, the residue at position 37 (Fig. 2) that is shown to bind glutamate in GluCl_Cryst_ is not predicted to bind glutamate in GluCl*Ac*2 (Fig. 3A and B). Likewise, the residue at position 93 (Fig. 2, surrounded by a red rectangle) aligns with a proline in GluCl_Cryst_ that was not found to bind glutamate in that receptor, whereas the arginine found at that position in GluCl*Ac*2 is predicted to bind to the α-carboxyl group. Although in both receptors the α-carboxyl group is involved in a salt bridge with an arginine, these two arginines - R37 in GluCl_Cryst_ and R135 in GluCl*Ac*2 (positions 37 and 93) - are neither aligned (Fig. 2), nor do they belong to the same subunit (Figures 3 and 4). Secondly, there is no equivalent in GluCl_Cryst_ for Y96 (position 54, surrounded in red in Fig. 2) which is predicted by the homology model for GluCl*Ac*2 to make a side-chain hydrogen bond with the α-carboxyl group and a hydrophobic contact with the backbone of glutamate (Figures 3 and 4).

#### Examination of GluClα2b in light of the GluCl_Cryst_ binding pocket

It should be recalled (see Methods) that the alpha subunit from which GluCl_Cryst_ was developed (GluClα1, accession # AAA50785) does not respond to glutamate without prior activation by ivermectin [12]. Thus it was not an adequate subunit for either evaluating the effects of mutations in the nematode receptor or for testing the sensitivity of that receptor to other agonists. Consequently, we selected another alpha subunit of the nematode receptor (GluClα2b, accession # CAA04170) which has the same binding mode as GluCl_Cryst_, but which responds directly to glutamate [32]. While all of the glutamate binding residues are conserved in the two nematode sequences (not shown), the present study identifies, at position 93, a critical arginine in GluCl*Ac*2 (see R135 in Figures 3 and 4) that aligns with a proline (P93) in GluCl_Cryst,_ but with a glutamine in GluClα2b (Q167; not shown). Simulations showed that this difference in the residue at position 93 in the two nematode receptors does not change their binding properties. We have therefore used the structure of GluCl_Cryst_ to elucidate the effect of mutations in GluClα2b.

### Site-directed-mutagenesis

#### The Mutations in the GluCl*Ac*2 receptor and their effects on the response to glutamate

**Figure 5:**
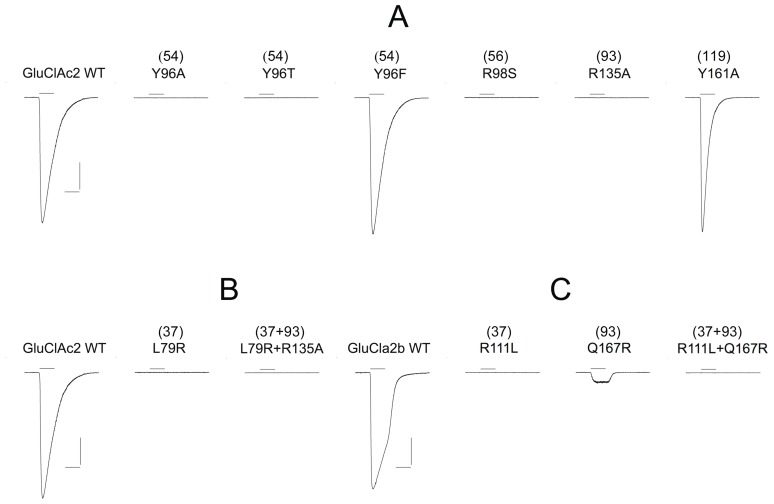
Responses to 1 mM glutamate in both WT and mutated GluCl*Ac*2 and GluClα2b receptors. A. Mutations in four binding residues of GluCl*Ac*2: Y96, R98, R135 and Y161 (positions 54, 56, 93 and 119). B. Mutations in GluCl*Ac*2 of residues L79 alone and L79+R135. These two residues are found at positions 37 and 93 (see Figs. 3 and 4). Only one of the two (R135) belongs to the GluCl*Ac*2 binding pocket (see the R135A mutation alone in A). C. Mutations in GluClα2b of residues R111 and Q167 found at positions 37 and 93, respectively: R111 alone, and R111+Q167 in a double mutation. Only one of the residues (R111) belongs to the GluClα2b binding pocket. Calibration: A: 1 sec, 500 pA; B: 1 sec, 500 pA; C: 1 sec, 1000 pA.

The homology model of the GluCl*Ac*2 binding pocket predicts that glutamate binds to five of the same residues to which it binds in the nematode binding pocket, as well as to a sixth “similar” residue. The alignment in Fig. 2 shows that these residues are found at positions 56, 121, 150, 151, 198 (green background in Fig. 4), with the sixth residue at position 201 (yellow background in Fig. 4). These positions correspond to residues R56, S121, S150, Y151, T197 and Y200 in GluCl_Cryst_, to residues R130, S195, S224, Y225, T271 and Y274 in GluClα2b; and to residues R98, S163, S192, Y193, T238 and F241 in GluCl*Ac*2. The homology model also predicts that, in GluCl*Ac*2, glutamate binds to three additional residues which are not included in the binding mode of the nematode receptor: Y96, R135 and Y161 at positions 54, 93 and 119, respectively (shown in Fig. 2 surrounded by a red rectangleand in Fig. 4 on a red background). Mutations were performed on four of the above residues in the GluCl*Ac*2 receptor: Y96, R98, R135 and Y161. Figure 5A reveals the differential importance of the mutations of these residues for the response to glutamate in GluCl*Ac*2. As was mentioned above, both of the WT receptors respond to 100 µM glutamate, and here it can be seen that there is no response in many of the mutations to a 10 fold higher concentration (i.e., 1 mM).

**Figure 6:**
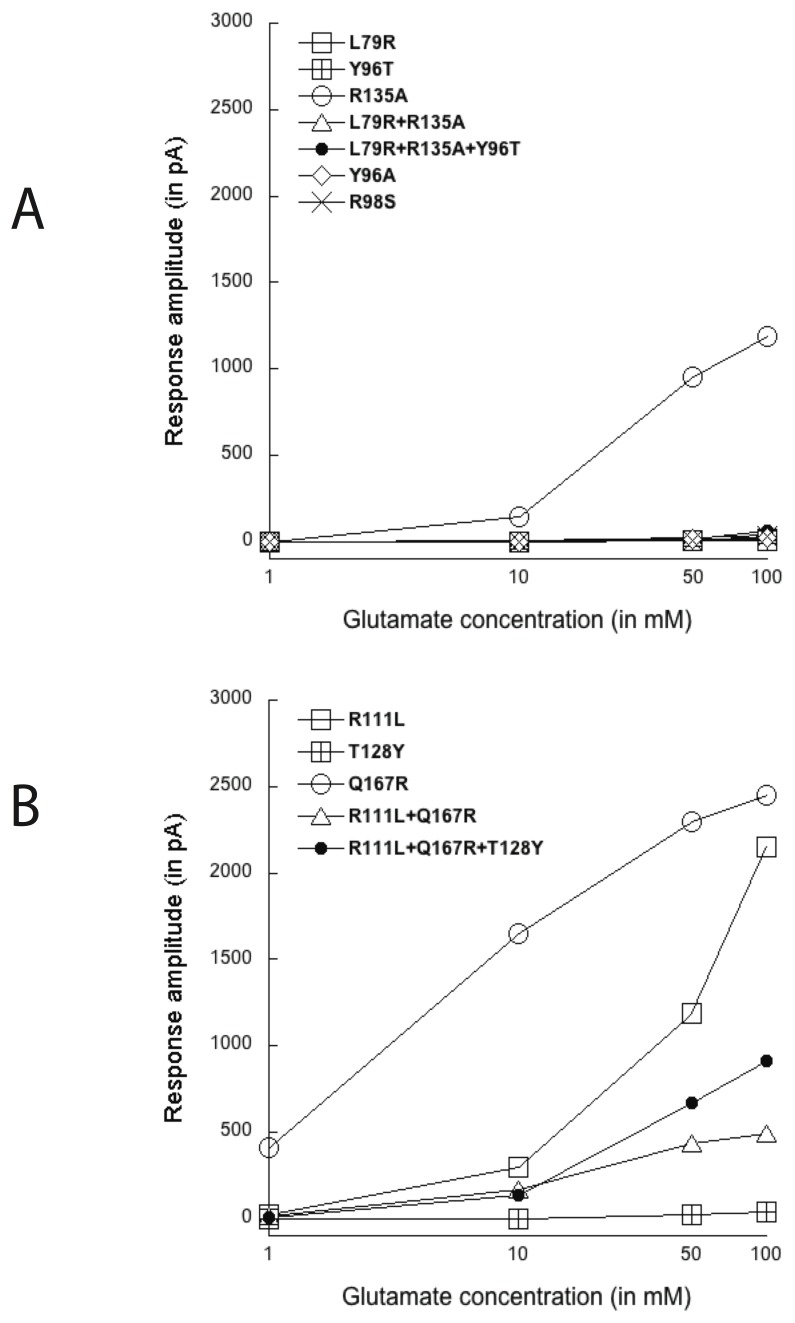
Responses of mutated GluCl*Ac*2 and GluClα2b receptors to increasing concentrations of glutamate. A: Responses (in pA) of seven mutated GluCl*Ac*2 receptors to 1, 10, 50 and 100 mM glutamate. B: Responses of five mutated GluClα2B receptors to 1, 10, 50 and 100 mM glutamate.

Unlike most of the mutations, that of residue Y96 (position 54, see Fig. 2) to a phenylalanine caused no change in the response to glutamate (Fig. 5A, Y96F). On the other hand, when that same residue was mutated to either an alanine or a threonine (the latter of which is the residue at the same position in the nematode receptor) the response was completely eliminated. As can be seen in Fig. 6A, the Y96A and Y96T mutations rendered the receptors unresponsive even to a 100 mM glutamate concentration.

As can be seen in Fig. 5A, the response to 1 mM glutamate was also lost when the residue R98 (position 56) was replaced by a serine (R98S) or when R135 (position 93) was replaced by an alanine (R135A). However the persistence of the deleterious effect of the two mutations at higher concentrations differed significantly. Whereas, even at 100 mM glutamate, there was no sign of a response from the R98S mutation, in the receptor with the R135A mutation a small response was obtained with 10 mM, and a robust response was obtained with 50 and 100 mM glutamate (Fig. 6A).

The residue Y161 (position 119 see Fig. 2) was found not to be critical for binding glutamate since its mutation to an alanine failed to cause any noticeable change in the response to glutamate, as can be seen in the last record of Fig. 5A.

#### Modifications of the non-binding residues in one receptor that are aligned with a binding arginine in the other receptor

We also mutated, in each of the receptors, a non-binding residue that aligns with a critical binding arginine in its homolog. Thus, the non-binding leucine L79 in GluCl*A*c2 (position 37, Fig. 2) was mutated to an arginine. This mutation resulted in adding a supplementary arginine to the binding pocket, and, as can be seen in Fig. 5B (L79R), this additional arginine eliminated the response to 1 mM glutamate - as it did at all concentrations of glutamate (see Fig. 6A).

In GluClα2b, in contrast, the addition of an arginine at position 93 by the Q167R mutation (Fig. 5C) only reduced, but did not completely eliminate, the response to 1 mM glutamate which recovered at higher glutamate concentrations (Fig. 6B).

#### Mutations in both receptors designed to evaluate the significance of “non-aligned”, binding arginine residues

We also verified the significance of each of the “non-aligned” binding arginine residues. We first mutated the residue R135 (position 93, see Fig. 2) of GluCl*Ac*2 to an alanine. As can be seen in Fig. 5A, that non-aligned arginine affects glutamate binding in the *Aplysia* receptor since the response to 1 mM glutamate was eliminated. However, it is considerably less effective in inhibiting the response to 10, 50 and 100 mM glutamate than are the other GluCl*Ac*2 mutations (see Fig. 6A).

The corresponding non-aligned, binding arginine found in the GluClα2b receptor at position 37 was mutated to a leucine (the amino acid found in the homologous position in GluCl*Ac*2). This arginine mutation affected glutamate binding (see R111L in Fig. 5C), although a greatly reduced response to 1 mM glutamate could sometimes be obtained and, as can be seen in Fig. 6B, this mutation did not impede a robust response to glutamate at higher concentrations.

Double mutations were made of the residues at positions 37 and 93 in both GluCl*Ac*2 and GluClα2b. These mutations introduced a switch of the positions of their non-common arginines. This was done in order to evaluate the effect of the position of the second arginine on the ability of each of the receptors to bind glutamate.

The switched positions in GluCl*Ac*2 eliminated the response to glutamate at all concentrations (see L79R+R135A in Figures 5B and 6A), whereas in the cells bearing the corresponding double mutation in GluClα2b receptor (see R111L+Q167R) higher concentrations of glutamate elicited responses in the 100’s of pA (Fig. 6B).

#### Triple mutations in the two receptors

Finally, triple mutations of the amino acids either binding the α-carboxyl group or aligned with amino acids in the second receptor that do so bind were also performed (not shown in Fig. 5). As with the double mutations, this complete exchange of the three residues in positions 37, 54 and 93 (see Figures 3B and 4) rendered GluCl*Ac*2 unresponsive at all concentrations of glutamate (Fig. 6A), whereas, once more, the corresponding exchange of residues in GluClα2b did not impede the appearance of responses in the hundreds of nA to higher glutamate concentrations.

#### Summary of the effects of the mutations in both receptors

Whereas six of the seven mutated GluCl*Ac*2 receptors remained essentially unresponsive to all concentrations of glutamate (highest average response to 100 mM glutamate in the 6 receptors was 62 pA), four of the five mutated GluClα2b receptors responded robustly to 10, 50 and 100 mM glutamate. In both receptors the mutation of the residue at position 93 (Fig. 6) was the least damaging. In GluCl*Ac*2, the receptor bearing that mutation (R135A) was the only one of the seven mutations that showed a response to glutamate at higher concentrations, and its behavior as a function of glutamate concentration (see Fig. 6A) was found to be significantly different from all of the other GluCl*Ac*2 mutations grouped together for a 2-way ANOVA (p. <.0001).

Of the mutations performed on GluClα2b, as in the corresponding mutation in the *Aplysia* receptor, that of the residue at position 93 (Q167R) showed robust responses at increasing glutamate concentrations. However, unlike in the *Aplysia* receptor, responses to 10, 50 and 100 mM could be seen for all of the mutations in GluClα2b except for the T128Y mutation which, like six of the mutations in GluCl*Ac*2, remained essentially unresponsive even to high glutamate concentrations (Fig. 6B). The responses seen as a function of glutamate concentration in the five mutated GluClα2b receptors were shown to differ significantly (2-way ANOVA, P<.0001). Of the five mutated nematode receptors only the comparison of the R111L+Q167R and R111L+Q167R+T128Y mutations failed to yield a significant difference (2-way ANOVA, p = .37).

#### The effect of the mutations on the response of the two receptors to GABA

In the two mutated GluCl*Ac*2 receptors for which there was no reduction in the response to glutamate (Y161A and Y96F at positions 119 and 54, respectively) the amplitude of the responses to 1 and 10 mM GABA likewise remained unaffected (not shown).

For the R135A (position 93) mutation there was a shift in sensitivity to both glutamate and GABA, with the threshold response for glutamate shifting from 1 to 10 mM, and that of GABA from 10 to 50 mM. For all of the other mutations in GluCl*Ac*2 there was a total disappearance of the response to GABA as there was for the response to glutamate. None of the mutations in the GluClα2b receptor transformed it into a GABA-sensitive receptor – even when tested with a 100 mM concentration of the agonist.

### Justification, in light of the model, for the effects of the mutations

#### Y161 is not essential for the binding of glutamate in GluCl*Ac*2

As was mentioned above, mutating Y161 (position 119) to an alanine did not affect the activity of the receptor (Fig. 5A). Indeed, in GluCl*Ac*2 as in GluCl_Cryst_, the γ-carboxyl group of glutamate is also interacting with an arginine, a serine and a threonine (at positions 56, 121 and 198, respectively; see Fig, 3 A and B and Fig. 4). Hence, it is not surprising that the removal of this additional interaction with Y161 fails to significantly disturb the binding of glutamate to the Aplysia receptor. Furthermore Y161 is binding the same beta sheet of the receptor as does S163 and is thus not providing an interaction with an independent secondary structure.

#### The role of the two arginines that are not shared by the two receptors

As can be seen in Fig. 5, both the R135A mutation in GluCl*Ac*2 (position 93) and the R111L mutation in GluClα2b (position 37) eliminate the response to 1 mM glutamate (Figures 5A and 5C, respectively), however both of these mutations show robust responses at higher glutamate concentrations. Neither of these two arginines, both of which bind the α-carboxyl group of glutamate, are essential for glutamate binding, however they are not equivalent. Indeed, in the double mutation in GluCl*Ac*2 (L79R+R135A; Fig. 5B) which moved the arginine from position 93 to position 37, the response was suppressed at all concentrations (see Fig. 6A), whereas in the corresponding double mutation in GluClα2b (R111L+Q167R), which moved the arginine from position 37 to 93, moderately-sized responses were already seen in the response to 10 mM glutamate (Fig. 6B).

From these data it can be seen that GluClα2b can accommodate the second arginine binding glutamate either at position 37 or 93. Supporting this statement, the Q167R mutation in GluClα2b, which adds a third arginine to the binding pocket, did not dramatically change the activity of the mutated receptor (see Fig. 5C). On the contrary, GluCl*Ac*2 cannot accommodate an arginine at position 37. Indeed, the single mutation L79R, which adds a third arginine to the binding pocket of GluCl*Ac*2 at position 37, essentially suppressed the response of the receptor at all glutamate concentrations (see Fig. 6A). The difference in the ability of the two receptors to accommodate the additional arginine can be explained by comparing the binding sites of the crystallographic structure of GluCl_Cryst_ with those presented in the homology model of GluCl*Ac*2, and by more precisely studying the position of the two arginines and their respective environments.

As can be seen in Fig. 3A and B, the two non-common arginines are not identically positioned relative to glutamate in the two receptors. In GluCl_Cryst_, the arginine at position 37 is located in the middle of the binding pocket and on the C subunit (see Fig. 3A and B), and pulls the α-carboxyl group towards the center of the binding pocket, whereas the arginine at position 93 in GluCl*Ac*2 (R135) is located at the far end of the binding pocket on the P subunit of the dimer, with the α-carboxyl group facing in the corresponding direction (see Fig. 3A and B). These differences result in a different glutamate bioactive conformation in the two receptors. The residues aligned with the non-common arginines are similarly positioned (L79 in GluCl*Ac*2 and P93 in GluCl_Cryst_, Fig. 3A and B). The difference in the effectiveness of the double mutation experiments results from the different environment of the residue at position 37 in the two receptors: in particular with respect to the residues at position 54 which are T54 in GluCl_Cryst_ and Y96 in GluCl*Ac*2 (see Figures 3A and 5B). The mutations of the two non-common arginines (R135 at position 93 in GluCl*Ac*2 and R111 at position 37 in GluClα2b) demonstrate that 1) in neither of the two receptors is the binding of an arginine to the α-carboxyl group of glutamate critical, 2) the non-common arginines define the orientation of the glutamate at the binding site, and 3) in neither receptor can one of the two non-common arginines substitute for the other.

#### The role of Y96 in GluCl*Ac*2

On the other hand, the Y96T mutant, like the Y96A mutant, showed essentially no response even to 100 mM glutamate, whereas the mutation Y96F did not affect receptor activation (see Fig. 5A). It can be concluded, thus, that the Y96 residue owes its major role in the binding pocket to the hydrophobic contact that its phenyl ring makes with glutamate. The stability of the link between Y96 and glutamate depends, however, on the hydrophobic contact that Y96 has with L79 (position 37). This latter hydrophobic support presumably orients Y96, making it possible for its phenyl ring to keep glutamate in an extended conformation (Fig. 3A and B). This hypothesis is consistent with the observed effect of the L79R mutation: indeed, an arginine’s side chain is longer than that of a leucine, therefore this L79R mutation would prevent Y96 from adopting the conformation that accommodates glutamate in the binding pocket. Thus, the link between L79 and glutamate is obtained via Y96 in GluCl*A*c2, whereas in GluClα2b, a direct interaction between R37 and glutamate is responsible for the stabilization between the β1 sheet of the receptor and the ligand. This situation results in different glutamate conformations in GluCl_Cryst_ and GluCl*Ac*2 characterized by a longer distance (d_1_) between amino and γ-carboxyl groups in GluCl_Cryst_ (4.9 Å) than in GluCl*Ac*2 (4.4 Å) (see [48] for d_1_ definition).

### Examination of the different pharmacological sensitivities of the Aplysia and nematode receptors in light of the model

As was seen in Fig. 1, differences in the responses of the nematode and *Aplysia* receptors were observed when they were exposed to GABA, β-alanine and taurine. These agonists activate and desensitize GluCl*Ac*2, whereas they have no effect on GluClα2b.

**Figure 7:**
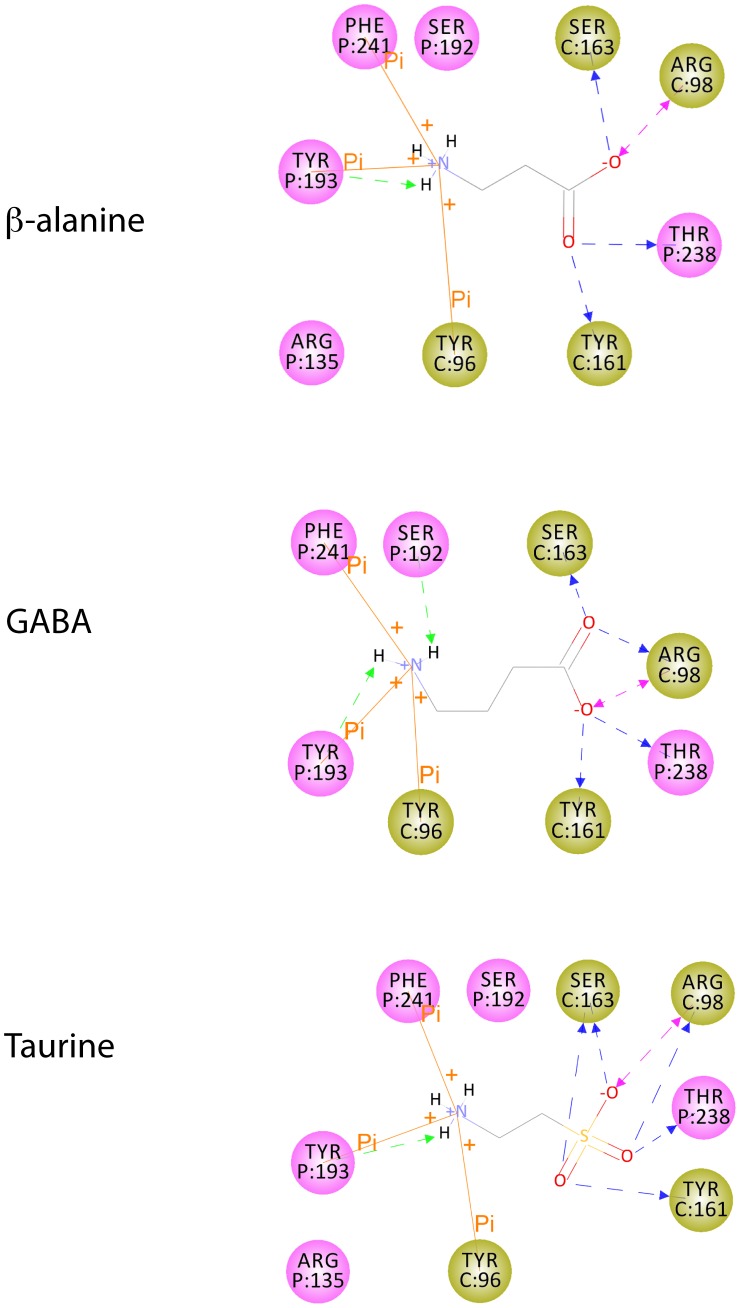
Similar binding modes for the three additional ligands in GluCl*Ac*2. 2D diagram representing the interactions between the binding pocket residues of the homology model for GluCl*Ac*2 and (A) β-alanine, (B) GABA and (C) taurine. Ligands are represented in lines. Only the polar hydrogens that are involved in interactions with the receptor are explicitly represented. Residues are depicted as circles in which the residue type, number and position (the latter in parentheses) are written on a colored background which indicates the subunit to which the residue belongs (see Fig. 3 and Fig. 4). Backbone and side chain hydrogen bonds are represented by green and blue arrows, respectively. Salt bridges are represented by purple arrows, π interactions are represented by orange lines. Atom colors as in Fig. 3B.

All three of these ligands lack the α-carboxyl group found in glutamate, and two of the three (β-alanine and taurine) are shorter than glutamate. The weaker potency of these agonists compared to glutamate can thus be explained by the loss of the interactions in which the α-carboxyl group was involved. Yet they still activate the receptor which is consistent with the mutagenesis data of R135A. Indeed the potency of GABA and β-alanine on the WT is similar to that of glutamate on the R135A mutant. Nevertheless, it is worth noting that even if these ligands lack the α-carboxyl group of glutamate, there remains a difference in the response of the two receptors to these ligands. We have already seen that glutamate adopts different bioactive conformations in the binding pockets of the two receptors, so the three ligands were docked in GluCl*Ac*2’s homology model to illustrate the structural explanation for these experimental observations. The results are shown in Fig. 7.

A first observation stemming from the dockings is that the γ-carboxyl group of GABA and β-alanine, and the sulfonate moiety of taurine bind to GluCl*Ac*2 with the same network of interactions as does the γ-carboxyl group of glutamate (R98, S163, T238). However, the lack of an α-carboxyl group in these agonists results in R135 no longer being involved in any interaction with the ligands. Nevertheless, as described above, this loss of interaction does not prevent the receptor activation but simply reduces the potency of the agonist.

Secondly, one can observe that as the carbon chain of the ligand is shortened, the α-amino group is closer to Y96 than it is when glutamate is docked. As can be seen in Fig. 7, the α-amino group of the three ligands is involved in a cation-π interaction with Y96. This new interaction, not present when glutamate is docked in GluCl*Ac*2, stabilizes the α-amino group of these ligands, and could partly compensate the loss of interaction with R135. This thus supports the experimental observation that GABA, β-alanine and taurine can weakly activate GluCl*Ac*2.

Finally, we can compare the organization of the binding pockets of GluCl*Ac*2 and GluCl_Cryst_ (and thereby that of GluClα2b) and notice that, structurally, Y96 in GluCl*Ac*2 and R37 in GluCl_Cryst_ are close together on their β2 and β1 sheets, respectively, and are on the same subunit (see Fig. 3A and B). Because of the short d_1_ distance in β-alanine and taurine, their α-amino group function is nearer to the arginine at position 37 in the nematode, thus creating a repulsion between the two positive charges which impedes the binding of these ligands. Moreover, this repulsion is screened by the α-carboxyl group in glutamate. The absence of this group in GABA, β-alanine and taurine suppresses the screening effect and allows the repulsion between the α-amino group of those ligands and R37.

#### Residues at positions 37, 54 and 93 appear to determine the pharmacology of other pentameric receptors much in the same way as they do that of GluCl*Ac*2

**Figure 8:**
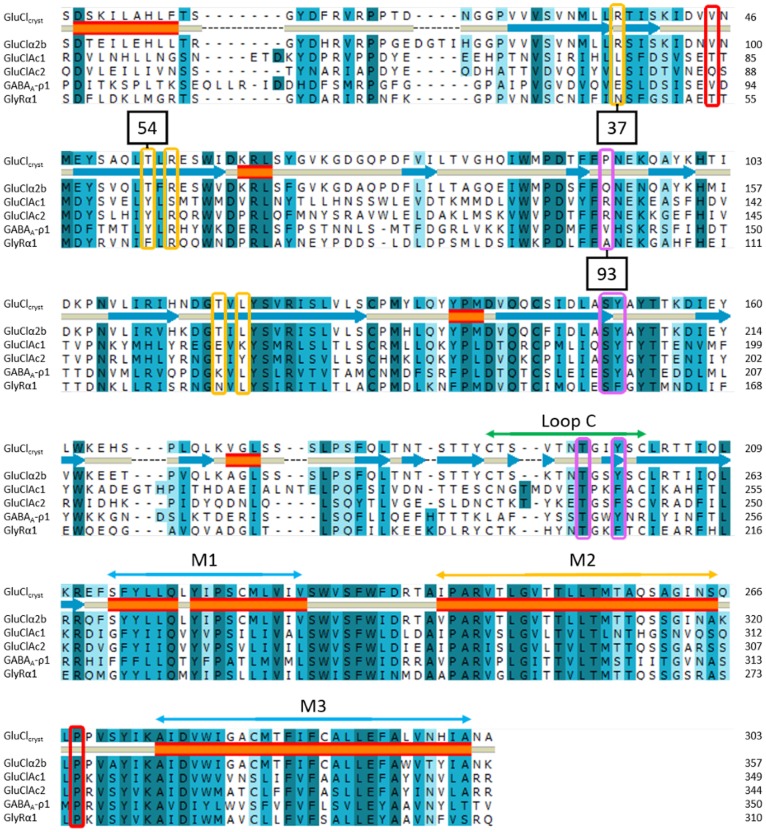
Alignment of the residues from the binding pocket region through the third transmembrane region of several ligand-gated ion channels. The alignment contains the sequences from four invertebrate glutamate-gated chloride channels (GluCl_Cryst_ and GluClα2b from *C. Elegans* and GluCl*Ac*1 and GluCl*Ac*2 from *Aplysia californica*), and those from two vertebrate receptors: the glycine receptor Glyα1 from *Rattus norvegicus* (accession #CAC35979 ) and the GABA receptor from *Homo sapiens*, GABA_A_-ρ1 (accession #EAW48558). The second line represents the secondary structure of GluCl_Cryst_: the blue arrows represent β-sheets; the orange tubes, the α-helices. Loop C and helices M1, M2 and M3 are indicated above the alignment. Positions 37, 54 and 93 are indicated in black boxes. Identical, strongly similar and weakly similar residues are highlighted, respectively, in dark blue, medium blue and light blue. Residues of interest for the binding of glutamate that were unveiled in this article are surrounded by violet rectangles when the residues are on the Principal face, and are surrounded by yellow rectangles when on the Complementary face. Residues surrounded by a red rectangle are involved in the opening/gating mechanism of the ion channel. The importance of a conserved proline in the M2–M3 extracellular loop will be discussed in Figure 10.

GluCl*Ac*2 is not the only pentameric receptor that fails to be selective for its defining agonist. The GABA_A_-ρ receptor, in addition to being activated by GABA, is also activated by β-alanine and taurine, and the glycine receptor, like the GluCl*Ac*2 receptor, can also be activated by all three of the additional agonists [40]–[43]. Figure 8 provides us with an alignment of the two nematode receptors, the GluCl*Ac*2 receptor and the two vertebrate receptors. It indicates the residues that appear to be critical for determining the different pharmacological sensitivities of these five receptors.

**Figure 9:**
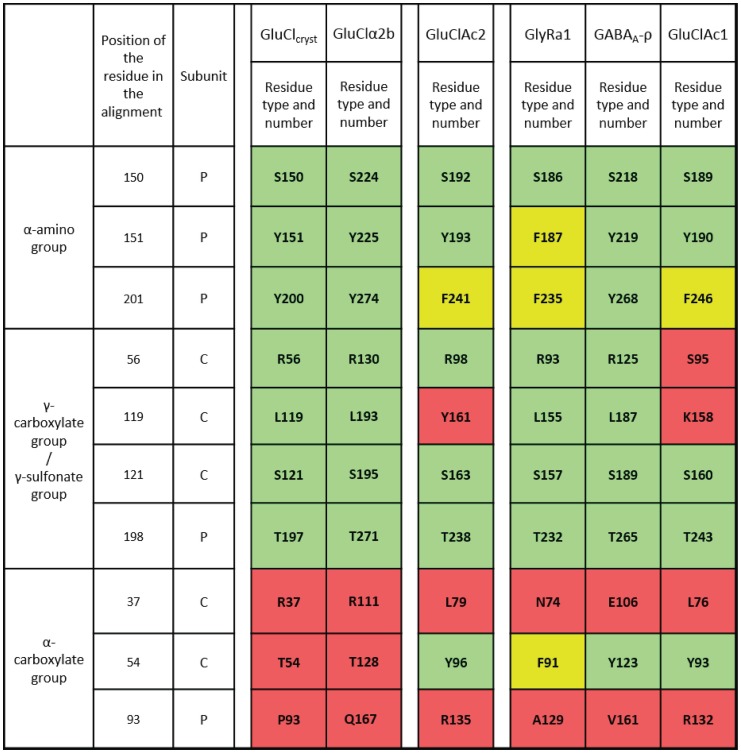
Corresponding numbers for aligned residues in GluCl from nematodes and *Aplysia*, Gluα1 and GABA_A_-ρ1. List of (1) the residues identified by the crystallized structure of the binding pocket of the two nematode receptors GluCl_Cryst_ and GluClα2b, (2) the residues predicted by the homology model for the binding pocket of GluCl*Ac*2 and (3) the corresponding aligned residues of four other receptors (GluClα2b, Glyα1, GABA_A_-ρ1 and GluClAc1) all of which were included in the alignment in Figure 8. The aligned residues fall into one of three categories defined by their known or anticipated site of interaction with the relevant ligand: α-amino group, γ-carboxylate/γ-sulfonate group, or α-carboxylate group. The aligned residues on a green background are identical, those on a yellow background are similar, whereas those on a red background are different. Note that at position 54, where the nematode receptors both have a non-binding threonine, there is a residue shown to be critical for agonist binding in GluCl*Ac*2 (Y96). The other *Aplysia* receptor as well as the glycine and GABA receptors all show an identical or similar residue to that of the GluCl*Ac*2 receptor at that position.

As can be seen in the alignment shown in Fig. 8, the two nematode receptors have an arginine at the position 37 which has been shown to be a critical binding residue for those receptors. None of the other four receptors indicated in Fig, 8 has a positively charged residue at that position. Likewise, at position 54, only the two nematode receptors have a threonine instead of the tyrosine or phenylalanine that are seen in the molluscan and vertebrate receptors (Glyα and GABA_A_-ρ) (see Fig. 9). That is, all of the receptors that bind ligands bearing only one acidic group (GluCl*Ac*2, Glyα and GABA_A_-ρ) have either a phenylalanine or a tyrosine at position 54, whereas GluClα2b, which is incapable of binding those supplementary ligands, does not contain a residue with a benzyl side-chain at position 54. The importance of such residues has already been demonstrated for the binding of glycine and GABA to the Glyα and GABA_A_-ρ receptors, respectively [46]–[47].

Residues binding the amino and γ–carboxyl groups of glutamate in GluCl_Cryst_ and Glu*Ac*2 are conserved in the glycine and GABA_A_-ρ receptors and bind the same type of functional groups in those receptors. These residues are found in green and yellow boxes in Figure 9 (positions 150, 151, 201, 56, 121, 198). We have seen that for the ligands that lack an α-carboxyl group, Y96 in GluCl*A*c2 interacts with the α-amino group. We can therefore hypothesize that the same residue plays a similar role in the Glyα and GABA_A_-ρ receptors (see Fig. 9 and [46]–[47]).

Finally, one can observe that unlike GluCl*A*c2, neither Glyα nor GABA_A_-ρ has a positively charged residue at position 93. Indeed, GABA does not have an α-carboxyl group and the carboxylate of glycine serves the role of the γ-carboxyl group of glutamate [46] (Fig. 9), hence no evolutionary pressure was put on this position to keep a residue that could bind with this moiety. This supports the hypothesis that no arginine is needed at position 93 to bind β-alanine, GABA and taurine in GluCl*A*c2. Thus, the similar broad pharmacology shared by GluCl*A*c2, Glyα and GABA_A_-ρ can be explained by the presence of a non-positively charged residue at position 37 and a residue with a benzyl side-chain at position 54.

#### Residues at positions 37 (β1-sheet), 54 and 56 (β2-sheet) are located on secondary structure elements delimiting a loop coupling the ligand binding and the gating domains

**Figure 10:**
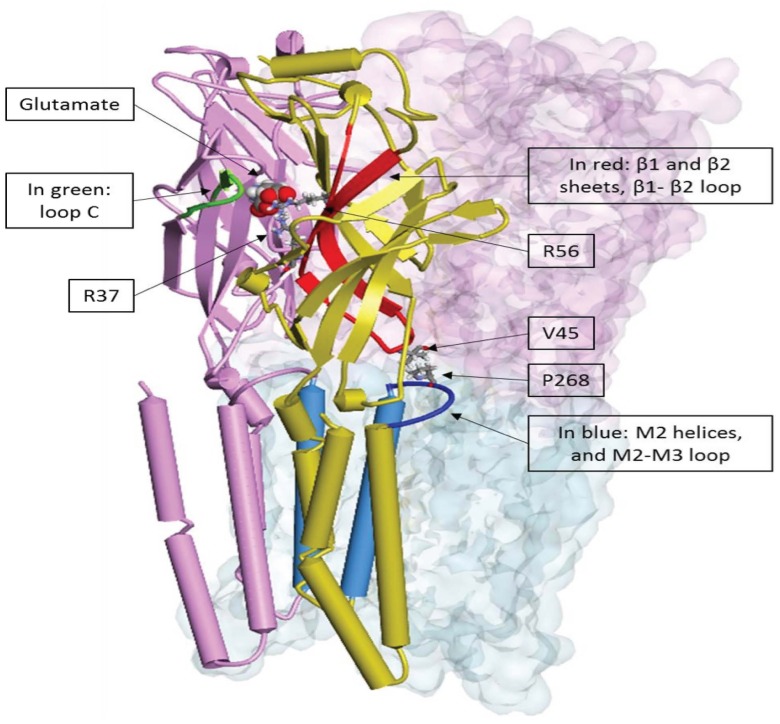
3D representation of the whole ion channel [49] of GluCl_Cryst_ (PDB code: 3RIF). Only two adjacent subunits are explicitly displayed, the three others are represented by the transparent surface. Alpha-helices are represented by tubes, beta-sheets by arrows (β1–β2 sheet in red, loop C in green, M2 helices and M2–M3 loop in blue). Principal and complementary subunits are colored, respectively, in violet and yellow. Glutamate is represented as CPK volumes, R37, V45, R56, and P268 are displayed in ball and stick. According to Calimet et al. V45 from β1–β2 loop and P268 from M2–M3 loop are involved in the gating mechanism. Interestingly the critical residues at positions 37, 54, 56 belong to β1–β2 sheets.

Calimet et al. [49], through long molecular dynamics, proposed a mechanism for the channel gating of GluCl_Cryst_. In this mechanism, the movement of loop C of the principal face (defined by residues between cysteines at positions 191 and 203 in Fig. 2 and indicated in Fig. 8) opens and closes the binding pocket. That movement is coupled with a global twist of the β sheet sandwiches of the extracellular domain, triggering the movement of the β1–β2 loop (between positions 40 and 48 in Fig. 2). This loop interacts with the loop between helices 2 (M2) and 3 (M3) of the transmembrane domain named M2–M3 loop (Fig. 8 and 10). V45 of loop β1–β2 can be either on one side or the other of P268 of M2–M3 loop, the transition being possible when loop C is opened. Finally, the position of loop M2–M3 influences the tilt of M2, and therefore the open or closed form of the channel (Fig. 10) [49].

The conclusions drawn above fit well with the proposed mechanism: residues at position 37 and 54, found to be essential and to determine the pharmacology of certain pentameric receptors, are located on the β1 and β2 sheets, as is the residue at position 56. The ligand must bind to the receptor before the closing of loop C which triggers activation of the receptor channel. In GluCl*Ac*2, the basic residue at position 56, along with the hydrophobic and aromatic residues at positions 37 and 54 (L79, Y96 and R98) make it possible, firstly, for a ligand with only one acidic group to interact with R98, and secondly for any positively charged or neutral atoms to interact with Y96. This explains the broader pharmacology of GluCl*Ac*2 compared to that of GluCl_Cryst_, for which the two basic residues (R37 and R56) only accept ligands such as glutamate that contains two acidic groups.

Furthermore, one can observe in Fig. 8 that the β1–β2 loop’s central residues are not conserved throughout all of these receptors, in particular at position 45 (V45 is the main residue interacting with the highly conserved proline of the M2–M3 loop P268 in GluCl_Cryst_ [49]). Thus, one can hypothesize that the exact mechanism of interaction and communication between the loops β1–β2 and M2–M3 is not the same in every receptor. This offers a possible reason for which the triple “reverse” mutation of GluCl*a*2b (R111L+Q167R+T128Y) failed to yield responses to the other three agonists to which the non-mutated GluCl*Ac*2 receptor responds.

### The fidelity of the two binding pockets to their respective phylogenetic groupings

#### Apparent orthologs of the GluCl*Ac*2 receptor found in other molluscs and annelids

**Figure 11:**
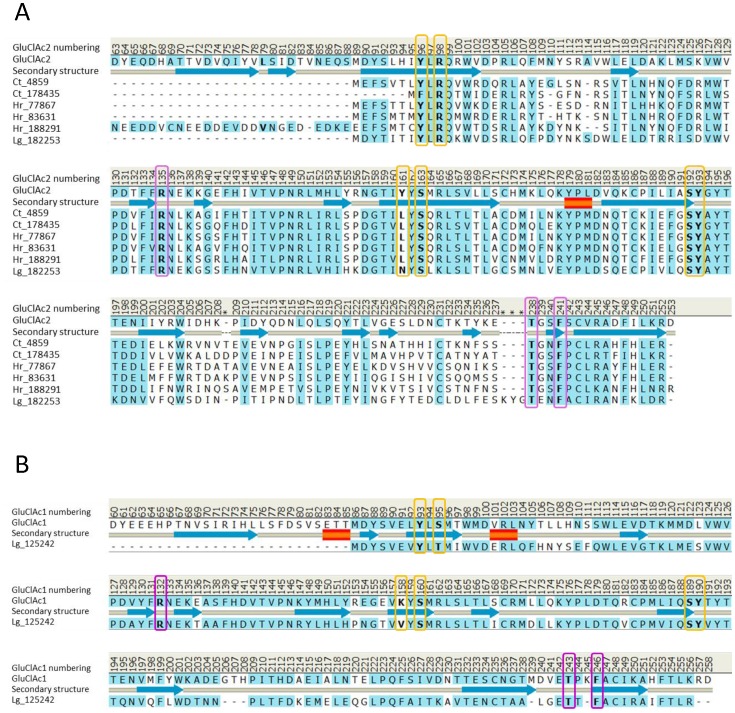
Orthologs of GluCl*Ac*2 (A) and GluCl*Ac*1 (B) obtained from three other lophotrochozoan species. A: Sequence alignment of predicted proteins from *Capitella teleta* (Ct), *Helobdella robusta* (*Hr*), and *Lottia gigantea* (*Lg*) with residues of the GluCl*Ac*2 receptor from *Aplysia californica* (*Ac*). The binding residues predicted by the homology model of GluCl*Ac*2 are bold and surrounded by either violet (P subunit) or yellow (C subunit) rectangles. Residue numbers from GluCl*Ac*2 are indicated above the alignment. B: Alignment of GluCl*Ac*1 and the second *Lottia gigantea* sequence (Lg_125242) which, like GluCl*Ac*1, fails to have an arginine at the position corresponding to the R98 of GluCl*Ac*2 (position 95 in GluCl*Ac*1). Furthermore neither Lg_125242 nor GluCl*Ac*1 have a tyrosine aligned with the position corresponding to Y161 of GluCl*Ac*2 (position 158 in GluCl*Ac*1).

Alignments of the N terminal region of the crystalized receptor with that of the other glutamate-gated chloride channels from *C. elegans*, as well as with the orthologous channels from other nematodes and arthropods, reveals that the binding mode as identified by crystallization represents that of both the nematode and the arthropod glutamate receptors. In contrast, an alignment of the N terminal region of the *Aplysia* (GluCl*Ac*2) receptor with 2-cys loop receptors from the nematodes and arthropods revealed no ecdysozoan receptors with a similar binding mode. On the other hand, a search of three lophotrochozoan genomes (*Lottia gigantea, Capitella teleta,* and *Helobdella robusta*) revealed two sequences from *Capitella*, three sequences from *Helobdella* and one sequence from *Lottia* that appear to be orthologs of GluCl*Ac*2 (see Fig. 11A).

It can be seen in Fig. 11A that none of the 2-cys loop sequences found in any of the three genomes, including the six predicted to be orthologs to GluCl*Ac*2, have a tyrosine aligning with Y161 of GluCl*Ac*2. However, since our mutagenesis experiments showed that the tyrosine was not necessary for obtaining a normal glutamate response, we have retained those six sequences as probable glutamate-gated chloride channels. Our mutagenesis experiments also showed that the mutation Y96F in no way affected the response of GluCl*Ac*2 to glutamate, so we have considered that the phenylalanine in that position in one of the selected sequences (Ct_178435) to be isofunctional with the tyrosine found in GluCl*Ac*2.

#### An apparent ortholog of the GluCl*Ac*1 receptor is found in the mollusc *Lottia gigantean*

It is interesting to note that whereas two orthologs were found in *Capitella* and three in *Helobdella*, only one GluCl*Ac*2 ortholog appears to exist in the *Lottia* genome. However, a second *Lottia* sequence was found that appears to be an ortholog of GluCl*Ac*1, the second glutamate-gated chloride channel cloned from *Aplysia* that was described by Kehoe et al. [14]. An alignment of GluCl*Ac*1 and its ortholog from *Lottia gigantea* is shown in Fig. 11B. It can be seen that neither the GluCl*Ac*1 receptor, nor its *Lottia* ortholog, has an arginine aligning with the critical R98 of the GluCl*Ac*2 receptor (see GluCl*Ac*1 residue number 95 in Fig. 9).

It was shown in Figures 5 and 6 that, under the 24 hour transfection constraints used here, a R98S mutation in GluCl*Ac*2 eliminated the glutamate response - even to a 100 mM concentration. Likewise, no response to 1 mM glutamate could be obtained from the expression of GluCl*Ac*1, which has a serine in that position in the WT. In the previous study on the *Aplysia* receptors [14], responses were obtained from GluCl*Ac*1 only when a much longer transfection period was used.

### Attempts to dock glutamate in the homology model of GluClAc1

An attempt to dock glutamate in the homology model of GluCl*Ac*1 (see Materials and Methods) was unsuccessful: when the docked glutamate was subjected to a molecular dynamics, it did not reach a stable conformation, thus indicating that the poor experimental activation of GluCl*Ac*1 by glutamate is reflected in the instability of glutamate in the binding pocket of GluCl*Ac*1’s model. However, a simple change in the two binding residues of GluCl*Ac*1 that were not aligned with the same amino acid in GluCl*Ac*2 permitted the stable docking of glutamate in the GluCl*Ac*1 homology model (see Materials and Methods). Those residues are found in GluCl*Ac*1 at position 56 (residue number 95, Fig. 9) where the serine in GluCl*Ac*1 was changed to an arginine as is found at the equivalent position in GluCl*Ac*2 (residue number 98, Fig. 9) and at position 119 (residue number 158, Fig. 9) where the lysine seen in GluCl*Ac*1 was changed to the tyrosine found at the same position in GluCl*Ac*2 (residue number 161, Fig. 9).

## Conclusion

In conclusion, the present study has revealed that although many residues of the glutamate binding sites are shared by the nematode and *Aplysia* glutamate-gated chloride channels, a few residues interacting with the α-carboxyl group of the ligand differ between the receptors. Two of the residues that differ appear to be responsible for the different pharmacological profiles of the two receptors. These residues are located at positions 37 and 54 (see Fig. 2).

In the *Aplysia* receptor the residues at these two positions are a leucine (L79) and a tyrosine (Y96). Y96 plays a significant role in permitting the docking of all four of the agonists by the promiscuous hydrophobic contacts its phenyl ring is able to make with the agonists. In addition, in the ligands lacking the α-carboxyl group, as the carbon chain of the ligand shortens the α-amino group becomes closer to Y96, thereby permitting a cation-π interaction between that residue and the α-amino group. Furthermore, the leucine at position 37 in the *Aplysia* receptor (as opposed to the arginine in the same position in the nematode receptor) assists in ensuring the binding efficacy of Y96 by its hydrophobic support of that residue.

In the nematode receptor, for neither the arginine at position 37 nor the threonine at position 54 is there a possibility of a direct binding of the other ligands, and in fact, the arginine at position 37 may well have a repulsive influence on the ability of those ligands to bind.

The fact that the GABA_A_-ρ and Glyα receptors have residues at positions 37 and 54 with the same characteristics as those found in GluCl*Ac*2, rather than with those of the nematode receptors, permit us to predict that the residues at those two positions are the ones responsible both for the failure of the nematode GluClRs to bind additional amino acids and, conversely, for the ability of GluCl*Ac*2 and GABA_A_-ρ and Glyα receptors to do so.
