# Correction: Molecular Determinants of Agonist Selectivity in Glutamate-Gated Chloride Channels Which Likely Explain the Agonist Selectivity of the Vertebrate Glycine and GABA_A_-ρ Receptors

**DOI:** 10.1371/journal.pone.0115788

**Published:** 2014-12-11

**Authors:** The PLOS ONE Staff

Figure 8 is incorrect. Please see a corrected version here.

**Figure 8:**
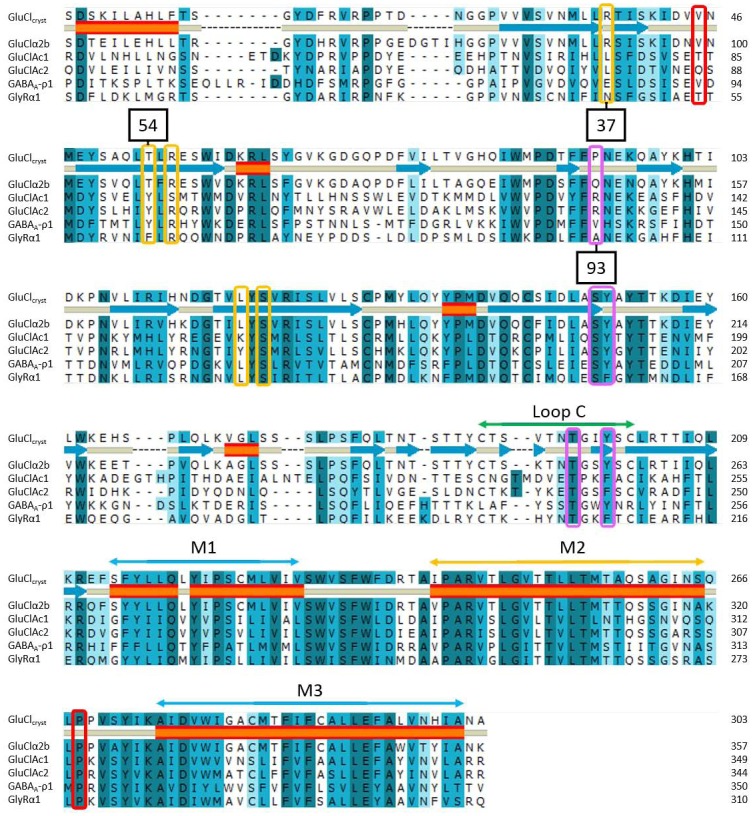
Alignment of the residues from the binding pocket region through the third transmembrane region of several ligand-gated ion channels. The alignment contains the sequences from four invertebrate glutamate-gated chloride channels (GluCl_Cryst_ and GluClα2b from *C. Elegans* and GluCl*Ac*1 and GluCl*Ac*2 from *Aplysia californica*), and those from two vertebrate receptors: the glycine receptor Glyα1 from *Rattus norvegicus* (accession #CAC35979 ) and the GABA receptor from *Homo sapiens*, GABA_A_-ρ1 (accession #EAW48558). The second line represents the secondary structure of GluCl_Cryst_: the blue arrows represent β-sheets; the orange tubes, the α-helices. Loop C and helices M1, M2 and M3 are indicated above the alignment. Positions 37, 54 and 93 are indicated in black boxes. Identical, strongly similar and weakly similar residues are highlighted, respectively, in dark blue, medium blue and light blue. Residues of interest for the binding of glutamate that were unveiled in this article are surrounded by violet rectangles when the residues are on the Principal face, and are surrounded by yellow rectangles when on the Complementary face. Residues surrounded by a red rectangle are involved in the opening/gating mechanism of the ion channel. The importance of a conserved proline in the M2–M3 extracellular loop will be discussed in Figure 10.
